# Oncologist and General Practitioner Perspectives of Shared Care for Colorectal Cancer Survivors: A Qualitative Study

**DOI:** 10.1002/pon.70223

**Published:** 2025-07-12

**Authors:** Karolina Lisy, Matthew Tieu, Claire Gore, Penelope Schofield, Raymond J. Chan, Jon Emery, Andrew Martin, Richard De Abreu Lourenco, Michael Jefford

**Affiliations:** ^1^ Department of Health Services Research Peter MacCallum Cancer Centre Melbourne Australia; ^2^ Australian Cancer Survivorship Centre Peter MacCallum Cancer Centre Melbourne Australia; ^3^ Sir Peter MacCallum Department of Oncology University of Melbourne Parkville Australia; ^4^ College of Nursing and Health Sciences Flinders University Adelaide Australia; ^5^ Psychosocial Oncology Program Peter MacCallum Cancer Centre Melbourne Australia; ^6^ School of Computing Engineering and Mathematical Sciences La Trobe University Melbourne Australia; ^7^ Department of Psychology Swinburne University of Technology Melbourne Australia; ^8^ Iverson Health Innovation Research Institute Swinburne University of Technology Melbourne Australia; ^9^ Matthew Flinders Professor of Cancer Care Systems and Policy Caring Futures Institute Adelaide Australia; ^10^ Centre for Cancer Research and Department of General Practice and Primary Care University of Melbourne Melbourne Australia; ^11^ National Health and Medical Research Council Clinical Trials Centre University of Sydney Sydney Australia; ^12^ Faculty of Health Centre for Health Economics Research and Evaluation University of Technology Sydney Sydney Australia

**Keywords:** cancer, cancer survivors, follow‐up care, models of care, oncology, primary care, shared care, survivorship, survivorship care

## Abstract

**Background:**

There is a growing body of evidence to support shared survivorship care. The shared care of colorectal cancer survivors (SCORE) randomised controlled trial (RCT) demonstrated that shared care is an appropriate and cost‐effective model. Understanding the perspectives of oncologists and general practitioners (GPs) who participated in SCORE will provide crucial insights to support wider implementation of shared care and adoption into clinical practice.

**Aims:**

To explore the experiences of oncologists and GPs who provided shared survivorship care for colorectal cancer survivors within the SCORE RCT, focussing on perceptions of acceptability and appropriateness of shared care, and facilitators and barriers to implementation.

**Methods:**

This qualitative descriptive study utilised semi‐structured interviews for data collection. Interviews were recorded and transcribed, and data analysed by hybrid deductive/inductive thematic analysis.

**Results:**

Interviews from 20 HCPs (13 GPs and 7 oncologists) were analysed. Seven themes were developed describing overall acceptance of the shared care model, the importance and challenges regarding bilateral communication between providers, mixed views on the need for GP training, and patients suitable for shared care. The need to support GPs with a direct hospital‐based contact person, as well as clear guidance on their role, was emphasised, as was the need for care coordination and logistical support.

**Conclusions:**

Our study offers novel findings regarding shared care from the perspective of participants who have direct experience with delivering the model. While shared care was broadly supported by both GPs and specialists, successful implementation requires agreed‐upon bilateral communication, clear guidance for GPs, and coordination support.

**Clinical Trial Registration:**

The Shared Care for Colorectal Cancer (SCORE) Trial is registered with the Australian New Zealand Clinical Trials Registry, ACTRN12617000004369p. Registered on 3 January 2017; protocol version 4 approved 24 February 2017.

## 1 Background

Colorectal cancer (CRC) is the third most common cancer worldwide, making up nearly 10% of all cancer diagnoses [1]. It was estimated that there were 1.4 million CRC survivors living in the United States alone, in 2022 [2]. Following primary treatment, CRC survivors typically participate in regular follow‐up care with their oncology provider(s) for a number of years, with follow‐up particularly focussing on surveillance for recurrence or new cancer [3, 4]. Such specialist‐led models of follow‐up are expensive and unsustainable in the context of a large and growing survivor population, and a limited health workforce [5, 6]. Survivors of CRC may also experience a range of physical, psychological, social and practical issues arising from their cancer, as well as comorbidities, that tend to be insufficiently addressed in a specialist‐led model, leading to survivors reporting unmet care needs and not achieving their optimal quality of life [4, 7, 8, 9].

As a result, there have long been calls for new approaches to follow‐up care [6, 10, 11, 12, 13, 14]. One alternative approach is shared care, a formalised collaboration between the specialist and a primary care provider, or general practitioner (GP), to provide follow‐up care. A growing body of evidence supports shared care as an effective and acceptable model, with equivalent clinical outcomes to usual, specialist‐led care [15, 16, 17, 18, 19]. Importantly, shared care is preferred by patients, leads to greater patient satisfaction, and costs less than standard specialist‐led follow‐up [15, 16, 17, 18]. Despite favourable evidence supporting shared care, specialist‐led care continues to be the dominant model for follow‐up care. Research supporting implementation of shared care has highlighted enablers to this model, including strong communication between providers, clarity regarding roles and responsibilities of each provider, care coordination, and information resources, such as survivorship care plans (SCPs) and practice guidelines [20, 21, 22].

The first randomised controlled trial (RCT) of shared follow‐up care for CRC survivors (SCORE) was recently completed, and, compared to usual care, demonstrated equivalent patient outcomes such as quality of life, unmet needs and satisfaction, superior adherence to recommended carcinoembryonic antigen (CEA) testing, no difference in recurrence rates or time to detection, and significant cost savings for the healthcare system [19]. Patients who experienced shared care also preferred it over specialist‐led care. To support wider and more sustained implementation of shared care, it is necessary to evaluate the SCORE intervention from the perspectives of participating providers.

The objective of this study was to explore the experiences and perspectives of health care providers (HCPs; both oncologists and GPs) who provided shared follow‐up care for CRC survivors as part of the SCORE RCT, examining the acceptability and appropriateness of the intervention, as well as facilitators and barriers to implementation.

## 2 Methods

### 2.1 Methodology

This qualitative descriptive study [23] was informed by the outcomes for implementation research defined by Proctor et al. [24] Consolidated criteria for reporting qualitative data (COREQ) [25] were adhered to. The SCORE intervention was delivered across five public hospitals in Melbourne, Victoria, and is described in full elsewhere [18, 19]. In brief, SCORE randomised CRC survivors to receive follow‐up care with their oncologist according to standard practice (usual care), or to shared care, where two hospital appointments were replaced with GP visits at three and nine months, with an additional GP visit soon after treatment completion. As part of shared care, patients received a survivorship care plan (SCP; shared with the GP and specialist), patient information booklet and DVD, and a common issues and concerns checklist; GPs additionally received clinical management guidelines. Ethical approval to conduct the study was received from Peter MacCallum Cancer Centre (Ref: HREC/72311/PMCC‐2020).

### 2.2 Participants

Eligible participants were oncologists and GPs who provided care for CRC survivors as part of the shared care intervention arm of the SCORE RCT. We used a mixture of purposive and convenience sampling, with an initial sample drawn from the full group to represent specialists across all five hospitals, and GPs from a diversity of metropolitan and regional areas. Invitations were sent to this initial sample, with those who responded offered interviews in order of availability. Participant recruitment and data collection occurred concurrently. Participants were recruited via email and letter, both containing information about the study, with follow‐up phone calls for non‐responders. Recruitment and data collection continued until two researchers (MT and KL), who had been independently reviewing interview transcripts, discussed and agreed that data saturation had been achieved, with data saturation defined as data collected in two consecutive interviews repeating what had already been expressed in the previous interviews [26]. Informed consent was provided verbally by participants and recorded prior to each interview.

## 3 Data Collection

Data were collected through semi‐structured interviews conducted via telephone or video call (Zoom or MS Teams) by one researcher (MT) from November 2022‐April 2023. MT is a research fellow, and an experienced qualitative researcher and interviewer. Separate interview schedules were created for GPs and oncologists based on provider‐level outcomes for implementation research defined by Proctor et al. [24] (Appendix 1) Interviews aimed to understand perspectives regarding acceptability and appropriateness of the SCORE shared care intervention, as well as providers' overall experiences, and perceptions of facilitators and barriers to providing shared care. Necessitated by the COVID pandemic, interviews also explored experiences and perceptions of providing follow‐up care via telehealth, which will be reported on separately. Interviews were recorded and transcribed verbatim using a professional transcription service.

### 3.1 Data Analysis

Transcripts were imported into NVivo (QSR International Pty Ltd) and analysed using combined inductive and deductive thematic analysis [27]. A broad and partial a priori coding framework was developed based on implementation outcomes [24] to include categories of acceptability of shared care, and perceived facilitators and barriers to shared care, with the intention of inductively coding data within these categories, and allowing for the creation of new categories emergent from the data. Transcripts were closely read and descriptive codes applied to the text, primarily by one researcher (KL). A subset of transcripts (10%) were reviewed and co‐coded by a second qualitative researcher (CG) to establish intercoder reliability. KL and CG independently coded randomly selected transcripts, then met to discuss, reconcile any differences and refine codes and emerging categories. Codes were analysed to explore patterns and similarities in meaning and to develop code categories which were then grouped together to develop themes. Themes and code categories were presented to the author team for further refinement and agreement. Results are presented using headings to describe the themes alongside illustrative participant quotes to substantiate the narrative.

## 4 Results

Overall, 27 specialists and 28 GPs were invited to participate, with 10 specialists (seven medical oncologists and three surgeons) and 13 GPs ultimately interviewed. Main reasons for non‐participation included no response to invitations and time constraints. This analysis includes interviews with 13 GPs and seven specialists (six medical oncologists and one surgeon) who participated in the shared care arm of the SCORE RCT. Thematic analysis led to the emergence of seven themes describing (1) acceptability and implementation; (2) communication between hospitals and primary care, (3) provider confidence, (4) training for GPs, (5) supportive resources for GPs, (6) suitable patients, and (7) care coordination and logistical support (Table 1).

**TABLE 1:** Summary of themes, with constituent code categories and exemplar participant quotes.

Theme	Code category	Participant quote
Acceptability and facilitators and barriers to implementation	Support for shared care	*‘I think every cancer patient should have [shared care]. Like a formal arrangement rather than just an ad hoc which often is what happens’.* GP2
	Benefits of shared care	*'…knowing that the patients got more complete holistic care and that they're seeing their GP, because they're probably attending to the other issues, chronic disease management and whatever, that we often worry are being neglected when they're coming to the cancer centre’.* SP1
	Implementation challenges	*‘We're shifting the work onto the general practitioners who are extremely overworked. There's a real sort of lack of GPs in rural and remote areas so… it would be very much making sure that the general practitioners could cope with the workload’.* SP7
	Facilitators to implementation	*‘I think that we probably need to make sure that the hospital team understands this is the model of care… that's an essential starting point, so the whole unit understands most people with colorectal cancer are managed by shared care, let's say. And so they expect it, and so they're not just going oh, there's some funny study that a few patients are on. You know, this is the usual practice’.* SP1
	Medicare (universal healthcare) and reimbursement	*‘Reimbursement. Yeah, that would be good to encourage some GPs'.* GP13
		*’[Reimbursement] would probably, you know, incentivise GPs that are not keen on doing it now or maybe to take it on board and start doing it’.* GP12
Communication between hospital and primary care	Two‐way communication is critical	*‘I think the GP involvement is critical in follow‐up and part of that is the communication backwards and forwards about the follow‐up and responsibilities and keeping the GPs informed about what's going on with the patient’.* SP7
	Inadequate communication from GPs	*‘I think our communication to GPs is reasonable; it's not great, it's reasonable, and coming the other way I think there's a deficit’.* SP5
	GPs satisfied with communication from hospital	*‘I was sent, you know, information about the patient and most of the time I had, you know, the updated follow‐up from the hospital and results and that come through to me in a reasonably speedy thing. Yeah, so just being kept in the loop and knowing exactly what was going on’.* GP4
	Challenges with communication	*‘The idea that the GP can get a scan done, like imaging, or* vice versa*, that the GP doesn't have access to our records and I don't have access to theirs is something that the patients find quite perturbing and, you know, a bit short‐sighted’.* SP7
	Role of technology to facilitate communication	*‘I would hope that with the increasing use of electronic health records or patient portals… where if the GPs are able to access our notes or health portals, that would be helpful but similarly, we have no way of looking at or accessing GP records’.* SP2
Provider confidence	GPs confident with SCORE intervention and their role	*‘I can't recall any situation where I thought I might have been out of my depth. So yeah, I felt confident’.* GP9
	Experience enhances GPs' confidence	*‘…doing that monitoring and I think once you do it a couple of times, as with any other you know, and then you can get used to it and do a bit better job in terms of—I think we did a good job but just be more comfortable with the whole process’.* GP1
	Communication enhances GPs' confidence	*‘The clinician may perhaps not feel confident in dealing with certain aspects, especially if they've not had experience or training in dealing with cancer patients… But ways round that would be to maybe have quick and easy access to a clinician at [cancer centre] where you can just get advice’.* GP9
	Specialists' confidence in GPs is mixed	*‘I don't have much confidence the GPs know exactly what they're looking for. I don't blame them because there's no consistent approach to it. There's no consistent approach even within one hospital, let alone multiple hospitals. Very, very fragmented, the care, the algorithms, guidelines’.* SP6
		*‘I feel confident as long as the GP says that they're confident and as long as there's a port of call for the GPs to refer back to us’.* SP4
Training for GPs	Perceptions that GPs would benefit from training	*‘I think I would have benefitted; I sometimes feel slightly inadequate in fully counselling the person because of—I'm not fully aware of the problem and what can be done for it, so I would personally find that very useful, you know, have some training’.* GP10
	Perceptions that training for GPs not required	*‘I personally believe that they don't necessarily need extra education and training but they do need guidance’.* SP1
	GPs learn from experience	*‘I've had over the years hundreds of patients who sadly have had colorectal cancer so I'm fairly up with what to be looking for but I'm sure I can be further educated’.* GP8
	Survivorship care training for GPs not feasible	*‘To expect the GP to be across the board for everything I think is unrealistic’.* SP9
	Training regarding CRC follow‐up actions	*‘So they won't know our usual surveillance follow‐up schedule, they won't know what tests are normally done at what surveillance follow‐up and so forth so in that way, yes, they do need some direction’.* SP6
Supportive resources for GPs	Recall of resources provided within SCORE	*‘I think the plan was clear; there was management guidelines… And it's good*. *I think there's a lot of information there about how to manage symptoms but I think in that there is useful information’.* GP1
	Clear guidance for GPs	*‘I think though that the GPs need to feel well‐supported, they need to have clear guidelines about what investigations need to be done, what potential side effects and treatments for those side effects’.* SP7
	Need a contact person in hospital	*‘Other than making sure there's like a known contact point so if something was going wrong that you knew that, yeah, ring this number, talk to this person directly, that's the only thing. Yeah, just specifying that a bit clearer. Yeah, I remember looking at the list and thinking okay, which one of these doctors do I call if I needed to’.* GP7
	Referral pathway back to hospital	*‘If the GP were to find something in their routine follow‐up that they were concerned about, they don't know if they need to be worried or not, then hopefully that coordinator or, you know, having a streamlined referral system back to the specialist clinics means that (a) the GP feels supported, (b) if there is a question that comes up from the GP's point of view where they don't know if they need to be worried or not, they can always ask for advice’.* SP4
	GPs feeling supported and part of the team	*‘It's making sure that GPs feel comfortable and supported by this and that they know that if there is a problem that we will then obviously be there to help’.* SP9
Suitable patients	Risk profiles and rare cancers	*‘I think the most important feedback you can get from me, that if there is a rare condition, rare syndrome, with high risk of cancer recurrence, I was not sure whether the shared care is a good idea or not’.* GP1
	Health literacy and self‐efficacy	*‘I believe the patient [is] also a big partner here and if the patient doesn't’ come and follow‐up then they're going to make it extremely difficult’.* GP5
	Patients with a trusted GP	*‘Patients need to have a trusted GP: We need to prepare GPs for that, we need to make sure people have got a GP so if they say I haven't got a GP or I don't like my GP, okay, we've got time to say this is how we deliver care… And so we should support people to have a GP who they're comfortable with so that when we are ready to think about shared care then that's ready’.* SP1
	Patient preference	‘*I think you'd have to talk to patients; I think they do get a certain satisfaction and comfort from seeing their specialist that they maybe don't get from seeing their GP’.* SP5
Care coordination and logistical support	Need for a care coordinator/administrative support	*‘I think that if we're going to do this then we need someone who can help coordinate the model, because at the moment we don't have a mechanism to do that’.* SP1
	Technology to support shared care	*‘Ideally supported by some technology which might be just SMS or whatever recall system that supports that. But at the moment, it's not enough’.* SP1
	Logistical challenges	*‘Occasionally correspondence might be delayed, probably due to admin issues perhaps, you know, if the patient, you know, it's gone to the wrong practice, if the patient was at a previous practice perhaps, they've changed and then it takes time to chase that up, you know’.* GP9

### 4.1 Acceptability and Facilitators and Barriers to Implementation

Shared care was acceptable to both GPs and specialists who expressed positive attitudes towards the model (‘*I think it's an excellent idea… I think everything we can do to have the GPs involved in the care and know what's going on so we're kept in the loop is really important’.* GP4). The benefit of reducing the load on oncology clinics was appealing to specialists (‘*It reduces some of the workload on our clinics… these are patients who might be quite quickly reviewed*’. SP2) and participants also articulated various benefits of shared care for patients, including more holistic care with a known GP (‘*They're usually having follow‐up by a clinician who knows them from a holistic perspective as well, so they're aware of the… family and social dynamics which usually has a significant component*’. GP9), and reduced travel and waiting times to see a GP compared with a specialist. Including a GP in survivorship care was also perceived to maintain continuity of care for the patient, and for patients without a trusted GP, entering into shared care may help them to establish a relationship with a new GP and therefore have the opportunity to receive ongoing care.

Doubts were raised about implementation of shared care in a real‐world setting, and how to translate an intervention within an RCT to standard care (‘*How well it's implemented is the issue probably*’. GP6). It was noted that making shared care the standard may require more rigorous processes to support the model (‘*I think if this was implemented in a serious fashion ongoing and everyone knew the direction, people would maybe make a bit more effort to make it work and develop some standards and protocols, SOPs [standard operating procedures] around the process’.* SP5). This may include standardized guidelines or protocols for CRC follow‐up care that would be shared across hospital and primary care settings, and resource allocation to support care coordination and dedicated staffing time, as well as policy directives that support shared care as the standard model. Further issues raised regarding implementation of shared care included primary care being at capacity (‘*If you want to, send us a new GP, because we don't have enough, then that would have helped*’. GP3), which was a particular concern in regional and rural areas, long wait times for patients to see GPs and additional cost for patients seeing a GP compared to their specialist at a public hospital. GPs further raised issues around reimbursement and the lack of billing codes for survivorship care as a major barrier to GP participation and broader implementation.

### 4.2 Communication Between Hospital and Primary Care

Two‐way communication between HCPs was cited as essential to support shared care, however, GPs and specialists expressed markedly different views regarding satisfaction with and expectations for communication between providers. GPs were satisfied with communication from the cancer centre regarding their shared patient, while specialists were dissatisfied with communication coming back from GPs (‘*I've never received any correspondence from a GP to tell me what their findings are. So we send all our correspondence to them, but we don't have equivalent correspondence from the GP back to the hospital to say that this is where they're at with their surveillance and things were normal or not’.* SP2). Specialists reported wanting information regarding their patients from GPs, however GPs did not articulate that regular communication to the hospital was part of their role. Most GPs expressed a need for a single direct contact person within the hospital, however indicated this was to ask questions, seek advice or report concerning results/symptoms, rather than a routine report back to the specialist.

It was suggested that part of a GP's role within shared survivorship care should include regular, routine reporting of follow‐up care and results back to the specialist ('*I think what's missing was maybe… if I am seeing the patient then I should write a letter to the hospital as well about that appointment, that this has happened, even if it's normal’.* GP1) and that GPs be involved as part of the care team prior to treatment completion via multidisciplinary meetings or case conferences ('*To be part of the team, I think is a good thing’*. GP8).

On a practical level, both GPs and oncologists reported challenges in sharing patient information across different services with different IT systems and platforms. Having access to an integrated system, such as an electronic medical record (EMR) that would allow sharing of patient information may ease communication across settings and facilitate shared care (‘*If there was an easy‐to‐use platform, and this would have to integrate with the electronic medical records… Then we can see what they've done, they can see what we've done and have a tool for escalation there’*. SP6).

### 4.3 Provider Confidence

GPs stated they were confident in providing CRC survivorship care, specifically around CEA tests and the follow‐up that was stipulated in the SCORE protocol (*‘Based on what was requested, required, yeah, that seemed okay’.* GP7). There were several factors which supported their confidence, including having a contact person at the hospital (‘*Am I comfortable with providing that* *support? Yes, I am. Absolutely… if I don't know something or if I don't understand something or if something needs to be clarified, I'm just calling the specialist’*. GP12) and having experience with providing survivorship care (*‘I think it's just a new concept and being a GP, I wasn't familiar with it so I think it's just I was anxious and apprehensive… and I think once you do it a couple of times… then you can get used to it and do a bit better job’.* GP1). Limited experience in providing cancer follow‐up care reduced GP confidence, but may be overcome with support from the hospital (‘…*the clinician may perhaps not feel confident in dealing with certain aspects, especially if they've not had experience or training in dealing with cancer patients… But ways round that would be to maybe have quick and easy access to a clinician at [hospital] where you can just get advice’.* GP9).

Specialists' confidence in GPs providing follow‐up care was mixed, and was impacted by factors including working in isolation and GPs seeing few CRC survivors in their practice (‘*I don't [have confidence]—I've certainly had anecdotal cases where the GP has done something different and I thought was inappropriate… So there was a misinterpretation of the result and also an inappropriate scare for the patient based on that result. So I think GPs' understanding of cancer biomarkers, they just don't have the volume, don't understand how these things can move around’*. SP5). Similar to GPs, specialists were more confident in a GP providing follow‐up care when they knew GPs were provided a point of contact within the cancer centre, as well as clear guidance (‘*I think if they're given a plan of what needs to be done and also a message easily—either contact us or re‐refer the patient back into the system, then yeah’*. SP7).

### 4.4 Training for GPs

Attitudes towards additional training for GPs were also mixed, with GPs and specialists overall expressing different views. Many GPs felt they may benefit from additional training (*‘I think you can always learn new things so if you were to put in some kind of programme, albeit a non‐tedious one, that would always be helpful’.* GP8), while specialists felt training for GPs to participate in shared care was not required. Rather than training, many specialists indicated that supporting GPs with resources such as clear guidance and a hospital contact was sufficient (‘…*not educating but giving GPs a protocol or schedule to follow and most importantly, having a way to contact us in an expedited fashion’*. SP4).

Challenges with requiring training for GPs included staying abreast of rapidly advancing knowledge (‘…*a lot of the things that's going on in the hospital is a bit over the top for the GP to fully understand… it's advancing so quickly that we couldn't keep up with it’*. GP5). It was also considered unrealistic for GPs to undergo training when they may see very few patients for follow‐up care, particularly given follow‐up will vary depending on cancer type, stage and treatment received.

In keeping with the idea that GPs need support through provision of clear guidance and a contact person within the hospital, training may focus on exactly what the GP is required to do within a shared care arrangement (‘*But I think there would certainly have to be some education for the general practitioner about what to do and, very importantly, when to refer back into the system’*. SP7).

### 4.5 Supportive Resources for GPs

Regarding resources provided to GPs as part of the shared care intervention, management guidelines and SCPs was considered useful (‘…*having the plan, like the formal plan, like you provided was good’*. GP7), however, few GPs recalled other resources that had been provided to patients. Aligning with the findings around GP training, data indicated that an essential supportive resource is clear guidance for GPs regarding follow‐up care, when to refer back to the hospital, information regarding a patient's diagnosis and treatment, as well as any treatment side or late effects; this may be communicated in a SCP (‘*If the GPs are given a very clear, in black and white, what they need to do. I think most of it was done by the [survivorship] care plan… can be really useful’*. GP1).

A direct contact at the hospital was also considered essential by GPs and specialists alike. Participants described needing a direct contact person for advice, to communicate concerning results, or to refer a patient for hospital follow‐up. This may be a designated role such as a nurse coordinator, or by contacting the treating specialist or team directly (‘*Just having that one contact point so that… it's quite clear that if there's any concerns, ring and talk to this person and having that direct number would probably be helpful*’. GP10).

Participants were satisfied with the guidance provided within the SCORE intervention and clear on what their role in the shared care model was, with GPs emphasising surveillance for recurrent disease (‘*There was, I think, a chart to say at that appointment what the doctor's supposed to do, like a straight examination, tumour marker and blood test. That was helpful. And I think the job of the doctor was simple’.* GP1), however there were some points of confusion regarding expectations and roles of each provider (‘*It was just open ended… the way it was spelt out to me, I wasn't sure of the extent of my role’.* GP10).

### 4.6 Suitable Patients

Participants perceived that shared care would work for many, but not all, cancer survivors, with characteristics making a patient suitable including having early stage disease, low risk of recurrence and ability to self‐manage (‘*I think it also does rely on having a patient that's very proactive… I think if there was a patient who… was not able to navigate the healthcare system that well then it would be a bit difficult’*. SP2). Other factors included whether a patient had a good relationship with a known GP, and patient preference for the model of care. Providing education for patients around the role of their GP within a shared care team, and linking patients in with a GP if needed, were suggested to support implementation of shared care.

### 4.7 Care Coordination and Logistical Support

Logistical issues were raised with shared care in the context of the SCORE trial, such as doubling up on follow‐up appointments, not knowing what tests had been done by the other provider in a shared care team, not having test results in time for appointments, correspondence delays, patients changing GPs, or patients not attending GP follow‐up (‘*I felt that the hospital—a few times when I thought I'd be following her up, it had already been done by the hospital’.* GP4).

Participants indicated that a care coordinator or another dedicated role would be required to facilitate shared care (‘*I think at the very simplest level, just having someone in that coordinator role who is actually able to make the necessary changes to help coordinate patients' appointments will be quite crucial’*. SP2), and that technology may be helpful *(‘In the ideal world, an EMR would flag and it would say this person's on shared care, do not see them for another six months’.* SP1).

## 5 Discussion

Here, we explored the views of HCPs who participated in the SCORE RCT regarding shared follow‐up care for CRC survivors, with the view to support future implementation of shared care. Overall, results demonstrate provider acceptability of shared care for CRC survivors, while providing valuable insights regarding modifications to the model that may support effective and sustainable implementation in the future. A qualitative evaluation of shared care from the perspective of patients who participated in SCORE has been published previously [28], with some themes including the importance of two‐way communication between providers and perceived benefits of seeing a GP common across both studies.

A major strength of this study is the exploration of shared care from the perspective of both GP and specialist providers who have themselves delivered the model. Other literature has explored the views of GPs [29] and specialists [30] regarding primary care involvement in survivorship from a hypothetical perspective, and we note strong overlap with our findings from the SCORE intervention. These include concerns regarding communication between the two settings, need for care coordination, the breadth of knowledge required to in general practice vs. the specialized knowledge required to independently deliver comprehensive survivorship care, GPs already operating at capacity, and incorporating risk profile and patient preference into the model of care chosen.

Regarding training for GPs as a requirement for cancer survivorship care, mixed attitudes have been reported in the literature previously [20, 29, 30], which aligns with the mixed views shared by participants here. Most GPs in our study felt they would have benefited from some form of training, and that this would enhance both patient care and their own confidence in care provision, however specialists were more likely to emphasise supporting GPs, rather than training. While it may not be feasible to provide disease‐specific training for GPs, as indicated by participants in our study, perhaps general survivorship training for GPs that provides an overview of survivorship care and core competencies may be appropriate, with studies indicating benefit across outcomes including behaviour, knowledge and confidence [31].

Rather than training for GPs, the two most prominent facilitators of shared care articulated in this study were (1) GPs having a direct point of contact within the hospital, and (2) GPs having clear guidance regarding the care they are expected to provide. The need for clear guidance for GPs within a shared care model has been found previously [20, 32], however here these elements were considered so crucial to successful implementation of shared care that they alleviated perceptions of GPs needing additional training, and enhanced the confidence of both GPs and specialists in care provided to patients. Despite this importance, our evaluation indicates that within SCORE, neither of these elements were optimally implemented.

Bilateral communication was certainly emphasised by both GPs and specialists in this study as being critical to implementation of shared care, which is in keeping with extant literature [20, 21, 22]. What is novel here in the context of the evaluation of SCORE is visibility on where communication broke down within the shared care intervention. From the data, it was clear that while specialists communicated information regarding patients to GPs, and GPs were satisfied with communication from hospitals, GPs did not necessarily communicate information regarding patient appointments and results back to specialists. In the absence of clear and agreed‐upon communication methods as part of the SCORE intervention, the expectation to communicate back to specialists was not clear to GPs, and therefore did not happen. Participants in our study indicated that a shared EMR accessible by all HCPs involved in a patients care may offer a potential solution to facilitate rapid sharing of information across settings, without adding time burden to the provider [33].

Many GPs in our study articulated the need for a single contact person at the hospital, indicating that GPs were not clear on who to contact, despite being provided with contact information for the treating team within the SCP. This suggests that including contact details for multiple treating specialists within a SCP without any further guidance around communication may not be an effective approach to providing this information to GPs. To support future implementation of shared care, a critical component of the model must be agreed‐upon communication channels between GPs and specialists, with visibility on one point of contact whom to direct communication to, and what information is expected to be shared. Further, as communication between providers has also been identified by patients to support confidence in shared care [28, 34], a clear communication plan may enhance acceptability of shared care for patients.

One perceived and oft‐mentioned benefit of shared care is the ability to provide patients with holistic care that encompasses all of their needs [8, 12, 29]. Broader survivorship care for CRC survivors should address psychosocial and practical concerns, health promotion and lifestyle behaviours, and screening for other primary cancers and diseases, in addition to surveillance for recurrence and help with physical concerns related to cancer and it's treatment [3, 4]. However, we found that while GPs indicated they were clear on their role in CRC follow‐up care, they perceived their role to be focussed on surveillance for recurrence, with CEA testing heavily emphasised and other elements of holistic survivorship care not mentioned. This is consistent with other data indicating that surveillance for recurrence is considered as the most important aspect of follow‐up from the perspective of GPs [35]. Certainly, results of the SCORE RCT are in keeping with this observation; while delivery or receipt of holistic care were not measured within SCORE, no differences in unmet needs were observed between shared care and usual care arms, and there was greater compliance with CEA testing observed in the shared care group [19]. The vast majority of GPs interviewed here also did not recall seeing or using the ‘common issues and concerns checklist’, which was provided specifically to patients ahead of their GP visits to aid in identification of individual patient needs. This is consistent with patient data which indicated that patients either did not recall receiving or did not use the information resources provided to them [28]. Together, this suggests that to realise the expected gain of holistic care within a shared care model, guidance to GPs on wider survivorship care and their role in providing this must be explicitly stated, resources to support this be provided to GPs as well as to patients, and additional work around resource development specifically to support shared care may be needed.

Studies from the patient perspective have also indicated that patients may express doubts around GPs' cancer‐specific knowledge in providing follow‐up cancer care [34], and this includes in the patient evaluation of SCORE [28]. However, it is important to note that patients who expressed concerns with their GP's ability to provide survivorship care tended to be those who experienced usual care, with those in the shared care arm being more confident in care provided by GPs, which is similar to results reported elsewhere [36]. Participants in SCORE who experienced shared care tended to prefer this model compared to usual care [19]. Patient preference was cited as a key consideration regarding patient suitability for shared care, and while patient buy‐in and acceptance is of course critical, so is the need to prepare patients for shared care. Earlier evidence‐based guidance for delivering shared care developed by the authors addresses this [20, 21, 37], and includes introducing shared care early on in a patient's care pathway, providing education around the role of follow‐up care and the benefits of accessing care through their GP, and assisting patients to find a trusted GP if needed.

### 5.1 Implications

It is critical that future studies of shared care do more to set clear and agreed‐upon expectations and channels for communication between GPs and specialists. Future studies should also strengthen guidance to GPs around provision of wider survivorship care, and emphasise the use of supportive resources to aid in identification and discussion of holistic survivorship needs. On a practical level, shared care must be supported by a care coordinator role to identify patients suitable for or on shared care, to assist in managing appointments and to facilitate information sharing between providers; this may also be supported by IT systems, such as an electronic health record, if available. Finally, future work to implement shared care and support a shift from clinical trials to clinical practice may be assisted by identification of solid implementation strategies that address the barriers to shared care identified here and earlier [20, 21], by accessing known implementation tools [38].

### 5.2 Limitations

Results of this study must be considered in the context of important limitations. Australian health care operates across public and private (paid for by insurance) settings; SCORE was delivered in the public health care setting only, and results may not be generalisable to specialists seeing patients in private health care settings, or to countries with varying health care systems. Interviews were conducted more than one year following completion of SCORE; as such, HCPs may have had difficulty recalling some aspects of their participation.

## 6 Conclusion

Both specialists and GPs found shared care for CRC survivors to be acceptable and supported the model. However, some aspects of the shared care intervention delivered within the SCORE RCT require further attention, namely communication from GPs to specialists, clarity regarding the role of GPs in providing holistic survivorship care, and effective provision and use of supportive resources by GPs. Study findings emphasised the need for two‐way communication between providers, specifically the requirement of a hospital‐based contact person for GPs, and clear guidance for GPs as keys for successful implementation. Future work can improve on the shared care model by addressing these barriers.

## Author Contributions

Conceptualisation: Michael Jefford, Penelope Schofield, Jon Emery, Andrew Martin, Richard De Abreu Lourenco, Karolina Lisy. Data curation: Matthew Tieu, Karolina Lisy. Formal analysis: Karolina Lisy, Claire Gore. Funding Acquisition: Michael Jefford, Penelope Schofield, Jon Emery, Andrew Martin, Richard De Abreu Lourenco, Karolina Lisy. Investigation: Matthew Tieu, Raymond J. Chan. Methodology: Michael Jefford, Penelope Schofield, Jon Emery, Andrew Martin, Richard De Abreu Lourenco, Karolina Lisy. Writing—original draft: Karolina Lisy. Writing—review and editing: Karolina Lisy, Matthew Tieu, Claire Gore, Penelope Schofield, Raymond J. Chan, Jon Emery, Andrew Martin, Richard De Abreu Lourenco, Michael Jefford.

## Conflicts of Interest

The authors declare no conflicts of interest.

## Appendix 1 Shared Care Interview Guides

### Shared Care: GP

Preamble: This interview is part of a study exploring the implementation and delivery of shared survivorship care. You have been asked to participate because you had a patient who received shared care as part of the SCORE trial, and we are interested in your experiences of providing follow‐up care as part of the trial. There are no right or wrong answers; each person's experience and opinions are important and we are interested to hear what you think so please be as open and honest as you can. I would also like to reassure you that neither you nor your patient will be identifiable from this research. This interview will be audio‐recorded and transcribed verbatim, and the transcript of the interview will be de‐identified and used for data analysis. Do you have any questions about anything I have said?

We have previously sent you information regarding the study. Can I please confirm that you have received and read this information provide consent to be interviewed today?

Q: Thinking back to the start of the trial, do you recall receiving any information or supportive resources to assist you with the follow‐up care of your patient?

- What were they? (GP Clinical Management Guidelines)
- Were they helpful? Why/why not?
- Was there anything else that you would have liked to have been told or given to support you to care for your patient?

Q: Do you recall the patient bringing a SCP along to the first appointment?

- What did you think of the SCP? Was it useful? Why/why not?

Q: Do you recall the patient using a patient concerns list, which is a list of common symptom issues or needs, at your appointments?

- If yes, was this helpful? Why/why not?

Q: I'm now going to ask you some questions about your overall experience of providing cancer follow‐up care.

- What was good about it?/what worked well?
- What didn't work well?/Did you experience any challenges?
- Do you think there are any advantages to shared care?
- Do you think there are any disadvantages to shared care?
- Did you feel you had enough communication with the patients' oncology providers? Why/why not?
- Was it clear to you what your role in the patient's follow‐up care was? Why/why not?

Q: Did you feel confident in providing cancer follow‐up care?

- Do you feel you would have benefited from any additional training or education prior to participating in shared care?

Q: Overall, what do you think about shared care for cancer survivors?

Q: Would you change anything about the shared care model? What and why?

Q: Thinking about the future, would you be happy to provide cancer follow‐up care for others in the future? Why/why not?

Q: If shared care was to become a standard of care in the future, how do you think this could this be supported? (Prompt if not already raised: reimbursement for survivorship care).

Q: Now I'm going to ask you a few specific questions about telehealth. Do you remember if you conducted any of your follow‐up appointments remotely via phone or video call?

If NO: Skip to Q*.

If YES:

Q: Can you tell me about the types of phone or video call appointments that you had (Describe the platform used, how it was set up).

Q: What systems or technology did you have to put in place to facilitate seeing patients remotely?

Q: Is telehealth something that you/your practice offered prior to COVID?

Q: How did you/others determine patients that were appropriate for telehealth and patients that were not?

Q: Compared to seeing your patient in person for cancer follow‐up, how did you find the phone/video consult?

- What was good about it?/What worked well?
- What didn't work well?/Did you experience any challenges? (Prompts: administration, appointment scheduling, technology systems)

Q: Did you feel that you could provide the same level of care remotely that you do when seeing a patient in person? Why/why not?

Q*: Do you plan to offer telehealth to your patients in the future/post‐COVID? Why/why not?

- What would help you to offer telehealth to patients in the future?

Q: If telehealth was to become routinely offered in the future, how do you think this could this be supported?

Q: Is there anything else you would like to tell me about either shared care or telehealth that we haven't covered?

### Shared Care: Oncologist

Preamble: This interview is part of a study exploring the implementation and delivery of shared survivorship care. You have been asked to participate because you had a patient who received shared care as part of the SCORE trial. There are no right or wrong answers; each person's experience and opinions are important and we are interested to hear what you think so please be as open and honest as you can. I would also like to reassure you that neither you nor your patient will be identifiable from this research. This interview will be audio‐recorded and transcribed verbatim and the transcript of the interview will be de‐identified and used for data analysis. Do you have any questions about anything I have said?

We have previously sent you information regarding the study. Can I please confirm that you have received and read this information provide consent to be interviewed today?

Q: I'm going to ask you some questions about your overall experience of providing cancer follow‐up care that is shared with a patient's GP.

- What was good about it?/what worked well?
- What didn't work well?/Did you experience any challenges?
- Do you think there are any advantages to shared care?
- Do you think there are any disadvantages to shared care?
- Did you feel you had adequate communication with the GP? Why/why not?
- Were you clear in what the GPs role in the patient's follow‐up care was? Why/why not?

Q: Did you feel confident in the GP providing cancer follow‐up care?

- Do you feel GPs need any additional training or education prior to participating in shared care?

Q: Overall, what do you think about shared care for cancer survivors?

Q: Would you change anything about the shared care model? What and why?

Q: Would you be happy to provide shared cancer follow‐up care for future patients? Why/why not?

Q: If shared care was to become a standard of care in the future, how do you think this could this be supported?

Q: Now I'm going to ask you a few specific questions about telehealth. Do you remember if you conducted any of your follow‐up appointments remotely via phone or video call?

If NO: Skip to Q*.

If YES:

Q: Can you tell me about the types of phone or video call appointments that you had? (Describe the platform used, how it was set up).

Q: What systems or technology did you have to put in place to facilitate seeing patients remotely?

Q: Is telehealth something that you offered prior to COVID?

Q: How did you/others determine patients that were appropriate for telehealth and patients that were not?

Q: Compared to seeing your patient in person for cancer follow‐up, how did you find the phone/video consult?

- What was good about it?/What worked well?
- What didn't work well?/Did you experience any challenges? (Prompts: administration, appointment scheduling, technology systems)

Q: Did you feel that you could provide the same level of care remotely that you do when seeing a patient in person? Why/why not?

Q*: Do you plan to offer telehealth to your patients in the future/post‐COVID? Why/why not?

- What would help you to offer telehealth to patients in the future?

Q: If telehealth was to become routinely offered in the future, how do you think this could this be supported?

Q: Is there anything else you would like to tell me about either shared care or telehealth that we haven't covered?
